# Spindle wave in intracranial pressure signal analysis for patients with traumatic brain injury: A single-center prospective observational cohort study

**DOI:** 10.3389/fphys.2022.1043328

**Published:** 2023-01-09

**Authors:** Jun Zhu, Yingchi Shan, Yihua Li, Jiaqi Liu, Xiang Wu, Guoyi Gao

**Affiliations:** ^1^ Department of Neurosurgery, Shanghai General Hospital, Shanghai Jiao Tong University School of Medicine, Shanghai, China; ^2^ Shanghai Head Trauma Institute, Shanghai, China

**Keywords:** traumatic brain injury, prognosis, intracranial pressure, parameter, spindle wave

## Abstract

**Objective:** Intracranial pressure (ICP) monitoring is an integral part of the multimodality monitoring system in the neural intensive care unit. The present study aimed to describe the morphology of the spindle wave (a shuttle shape with wide middle and narrow ends) during ICP signal monitoring in TBI patients and to investigate its clinical significance.

**Methods:** Sixty patients who received ICP sensor placement and admitted to the neurosurgical intensive care unit between January 2021 and September 2021 were prospectively enrolled. The patient’s Glasgow Coma Scale (GCS) score on admission and at discharge and length of stay in hospital were recorded. ICP monitoring data were monitored continuously. The primary endpoint was 6-month Glasgow Outcome Scale-Extended (GOSE) score. Patients with ICP spindle waves were assigned to the spindle wave group and those without were assigned to the control group. The correlation between the spindle wave and 6-month GOSE was analyzed. Meanwhile, the mean ICP and two ICP waveform-derived indices, ICP pulse amplitude (AMP) and correlation coefficient between AMP and ICP (RAP) were comparatively analyzed.

**Results:** There were no statistically significant differences between groups in terms of age (*p* = 0.89), gender composition (*p* = 0.62), and GCS score on admission (*p* = 0.73). Patients with spindle waves tended to have a higher GCS score at discharge (12.75 vs. 10.90, *p* = 0.01), a higher increment in GCS score during hospitalization (ΔGCS, the difference between discharge GCS score and admission GCS score) (4.95 vs. 2.80, *p* = 0.01), and a better 6-month GOSE score (4.90 vs. 3.68, *p* = 0.04) compared with the control group. And the total duration of the spindle wave was positively correlated with 6-month GOSE (r = 0.62, *p* = 0.004). Furthermore, the parameters evaluated during spindle waves, including mean ICP, AMP, and RAP, demonstrated significant decreases compared with the parameters before the occurrence of the spindle wave (all *p* < 0.025).

**Conclusion:** The ICP spindle wave was associated with a better prognosis in TBI patients. Physiological parameters such as ICP, AMP, and RAP were significantly improved when spindle waves occurred, which may explain the enhancement of clinical outcomes. Further studies are needed to investigate the pathophysiological mechanisms behind this wave.

## 1 Introduction

More than 50 million people worldwide suffer from Traumatic brain injury (TBI) each year, and it is estimated that about half of the world’s population will have one or more TBIs in their lifetime (Maas et al., 2017). TBI is currently the leading cause of disability and even death among young adults (Sandsmark, 2016).

The increase of intracranial pressure (ICP) is undoubtedly one of the most noteworthy events closely related to prognosis among the changes in various physiological states caused by TBI (Hawthorne and Piper, 2014). ICP monitoring, as a technical means to reflect ICP status in real-time, is of great value in guiding the formulation of the treatment plan and prognosis judgment (Farahvar et al., 2012). The Brain Trauma Foundation recommends ICP monitoring for all patients with severe TBI and abnormal computed tomography (CT) scans (Carney et al., 2017). A large prospective study suggested that the use of ICP monitoring may be associated with a more intensive treatment approach and reduced 6-month mortality in severe cases with brain injury (34% vs. 49%, *p* < 0.0001), potentially improving long-term clinical outcomes (Robba et al., 2021). Although meta-analyses of the impact of ICP monitoring on mortality in patients with sTBI (severe TBI) have reached different conclusions, recent studies have been consistent and have shown significantly higher survival rates (Glushakova et al., 2020). Based on the above advantages, ICP monitoring is currently widely carried out in centers at all levels and shows an upward trend.

Currently, the focus of ICP monitoring is primarily on the average ICP value in clinical practice. It is convenient to judge the rise of ICP simply by the mean value of ICP. However, as clinical research progresses, using ICP mean values alone to guide clinical decisions shows many limitations (Hu et al., 2010; Di Ieva et al., 2013). Dai et al. (2020) provided a review and outlook on ICP clinical monitoring and proposed a guiding framework for future research in the area of ICP signal. Recently, the purpose of monitoring has evolved from simply reversing elevated ICP or impaired cerebral perfusion pressure (CPP) to extracting additional diagnostic and prognostic information by analyzing ICP waveform and waveform parameters to characterize the physiological state of the brain (Di Ieva et al., 2013; Nucci et al., 2016; Donnelly et al., 2019; Pérez-Sánchez et al., 2021).

In our pilot ICP signal analysis studies, a rarely described ICP wave with a spindle shape was observed. Therefore, we decided to investigate the morphology of the ICP spindle wave in patients with TBI. In addition, clinical significance of the spindle wave and changes in waveform parameters during the spindle wave were focused on in this study.

## 2 Methods

### 2.1 Clinical data

The clinical data of TBI patients admitted to the Department of Neurosurgery, Shanghai General Hospital, Shanghai Jiao Tong University from January 2021 to September 2021 were prospectively collected. The inclusion criteria were as follows: 1) Age 18–65 years old; 2) emergency admission due to acute closed craniocerebral trauma; 3) clear history of trauma; 4) cranial CT manifestations and clinical symptoms were consistent with the surgical indications for intraventricular ICP monitoring in the Brain Trauma Foundation guidelines (Carney et al., 2017). The exclusion criteria were as follows: known pregnancy or the presence of contraindications to ICP monitoring such as abnormal coagulation function and serious blood system related diseases. The study protocol complied with the ethical guidelines of the Declaration of Helsinki, and this study was approved by the Ethics Committee of Shanghai General Hospital, Shanghai Jiao Tong University School of Medicine (RA-2019-180). The informed consent of participants (signed by the patient’s representatives) was fully guaranteed and indicated in the ethical approval document.

### 2.2 Data sources and measurements

Baseline data such as gender, age, and initial Glasgow Coma Scale (GCS) score of TBI patients were collected at admission. The included patients underwent emergency surgery according to trauma guidelines, including borehole drainage or cranial hematoma removal, or even decompressive craniectomy. Meanwhile, they received ICP sensor insertion (Sophysa, France, or Johnson & Johson, USA) during the operation and returned to the neurosurgical intensive care unit after surgery. All patients received analgesia, sedation, mechanical ventilation and were managed according to cerebral perfusion pressure-oriented protocol (including analgesia, sedation, intubation, mechanical ventilation, dehydration therapy, etc.). The target postoperative CPP range for all patients was 60–70 mmHg, with a treatment ICP threshold of 20 mmHg. The presence or absence of a spindle wave was not used to guide management of treatment. ICP values and waveform parameters of all postoperative patients were continuously monitored and recorded by a data processing tool (Neuro Critical Care Data Processing Software, Hunan Haotongxiangju Medical Technology), and ICP-derived indices were calculated. The sampling rate is 125 Hz and data was sent to the online server for final analysis work.

Two commonly used ICP-derived indices, AMP and RAP, were included in this study for analysis. AMP is defined and calculated from time domain perspective as the trough to peak difference of the ICP pulse wave (Eide, 2006; Howells et al., 2012), and the conventional view holds that an increase in the ICP pulse amplitude implies a worsening of the cerebrospinal compensatory reserve (Avezaat et al., 1979). RAP is the calculation of the correlation coefficient between AMP and ICP mean values for 40 consecutive unit times (6s is a time unit), representing the cerebrospinal compensatory reserve (Czosnyka and Pickard, 2004; Di Ieva et al., 2013). When the RAP value is close to 0, there is no correlation between AMP and ICP, reflecting the large compensatory space in the brain. Conversely, when the RAP is close to 1, it means that it is in the steep right side of the pressure-volume curve, and the brain’s compensatory capacity decreases (Zeiler et al., 2018; Dai et al., 2020). Both elevated AMP and RAP indexes indirectly reflect a decrease in intracranial compliance (Howells et al., 2012).

The patient’s GCS score and length of stay in hospital were recorded at discharge. Glasgow Outcome Scale-Extended (GOSE) score was followed-up at 6 months after injury. It was at that stage that we retrospectively summarized and analyzed the ICP data of the enrolled patients. The presence or absence of the spindle wave was not referenced during treatment to show how spindle waves behave in the natural course of TBI as well as to reduce the influence of subjective factors. Since the spindle wave has been found in the pilot study to be a series of spindle waves that are distinct from the basal ICP wave, it can be easily distinguished and defined. Patients with ICP spindle waves were assigned to the spindle wave group and patients without were assigned to the control group. Besides, parameters during one segment of spindle waves were compared with those 30 min before and after the spindle wave segment in the spindle wave group. Data were averaged around all segments of the same case and ultimately used one value per case for the analysis.

## 3 Statistical analysis

A two-tailed *p*-value of 0.05 or less was used to define statistical significance. Statistical analyses were done using R (version 3.5.0), with R Studio (version 1.1.447) used as the implementation integrated development environment. Measurement data conforming to normal distribution were expressed as mean (M) ± standard deviation (SD), and Student’s t-test or one-way ANOVA was used for comparison between the two groups. Continuous variables that do not conform to the normal distribution were expressed as the median and interquartile range (IQR). Pairwise t-test was used for comparison among parameters before, during, and after the spindle wave in the spindle wave group, and alpha was adjusted to 0.025 according to Bonferroni correction. Enumeration data were expressed as cases or percentages, and the chi-square test was used for comparison between groups. Pearson Correlation Coefficient was used to describe whether there is a linear correlation between continuous variables.

## 4 Results

A total of 60 patients with closed TBI who met the criteria were included, including 41 males and 19 females. All patients enrolled received emergency intraventricular ICP sensor insertion, 47 of them underwent simultaneous decompressive craniectomy. Participants were between 26 and 84 years old with a mean of (53.52 ± 15.25) years old. The admission Glasgow Coma Scale (GCS) scores were between 3 and 14 points, with a mean of (8.02 ± 3.31) points. Among the 60 patients, we found that 20 cases had spindle waves (spindle wave group, 33%) and 40 without spindle waves (control group, 67%).

### 4.1 ICP spindle wave

**FIGURE 1:**
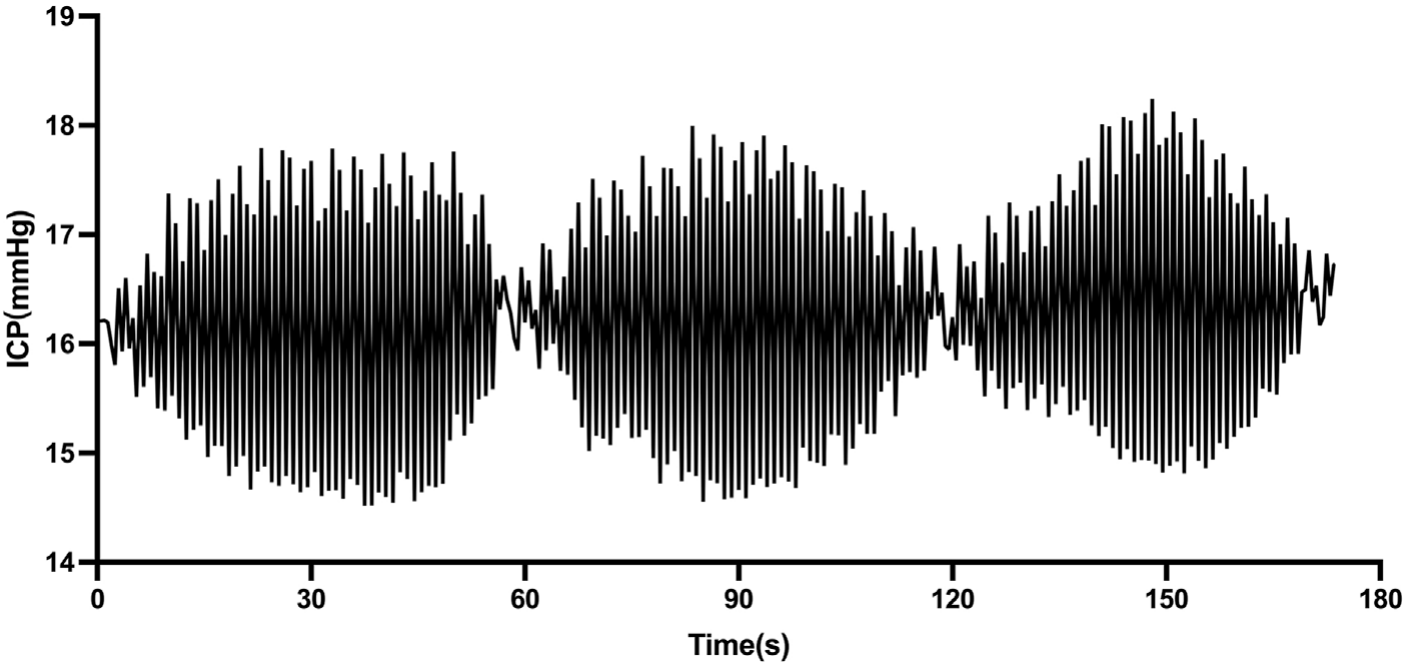
Morphology of spindle waves in patients with traumatic brain injury (TBI) (1 mmHg = 0.133 kPa).

The ICP spindle wave had a shuttle shape with wide middle and narrow ends (Figure 1). The average spindle wave amplitude (amplitude at the widest point of a single wave) in the spindle wave group was (4.51 ± 2.41) mmHg. The average duration of a single spindle wave in the spindle wave group was (21.35 ± 14.55) s and the average ICP at the time of the spindle wave was (18.11 ± 7.67) mmHg. The accumulated duration of spindle waves per patient in the spindle wave group throughout the monitoring process ranged from 137 to 588 min, with a mean of (314.55 ± 126.87) min. A total of 213 segments (a continuous segment from the appearance to the termination of the spindle wave) of the spindle wave in the spindle wave group were monitored, ranging from 6 to 18 segments per case, with an average of (10.65 ± 3.66) segments. The average duration of each segment was (31.63 ± 13.09) min.

### 4.2 Comparison of clinical data between the two groups

The clinical parameters of the two groups are shown in Table 1. There were no statistically significant differences between the two groups in terms of age (*p* = 0.89), gender composition (*p* = 0.62), and GCS score on admission (*p* = 0.73), which indicated that the two groups of patients were comparable in terms of patient population composition and disease severity. Eighteen patients in the spindle wave group and 29 in the control group underwent decompressive craniectomy, with no significant difference in composition between groups. The GCS score at discharge, the increment in GCS score during admission (ΔGCS, the difference between discharge GCS score and admission GCS score), and 6-month GOSE after injury were significantly higher in the spindle wave group than in the control group (12.75 vs. 10.90, *p* = 0.01; 4.95 vs. 2.80, *p* = 0.01; and 4.90 vs. 3.68, *p* = 0.04, respectively). No difference was found in the average length of stay in hospital between groups (*p* = 0.20). No difference was found in mean ICP values between groups during monitoring (*p* = 0.77). The correlation coefficient between AMP and ICP (RAP) was significantly lower in the experimental group than in the control group (*p* = 0.004).

**TABLE 1:** Comparison of baseline information between the two groups.

Variable	Spindle wave group (n = 20)		Control group (n = 40)	*p* value
Age (mean ± standard deviation, years)	53.10 ± 17.39		53.73 ± 14.30	0.89
Gender (male/female)	15/5		26/14	0.62
Decompressive craniectomy	18		29	0.19
GCS score on admission (mean ± standard deviation, points)	7.80 ± 3.52		8.13 ± 3.26	0.73
GCS score at discharge (mean ± standard deviation, points)	12.75 ± 1.97		10.90 ± 3.55	0.01
ΔGCS (mean ± standard deviation, points)	4.95 ± 2.19		2.80 ± 3.55	0.01
Length of stay in hospital (mean ± standard deviation, d)	18.65 ± 4.38		20.63 ± 7.27	0.20
6-month GOSE (mean ± standard deviation, points)	4.90 ± 2.10		3.68 ± 2.04	0.04
ICP (mean ± standard deviation, mmHg)	20.48 ± 7.54		19.88 ± 7.73	0.77
RAP (mean ± standard deviation)	0.21 ± 0.14		0.33 ± 0.15	0.004

GCS, is Glasgow Coma Scale; ICP, is intracranial pressure; GOSE, is Glasgow Outcome Scale-Extended; RAP, is the correlation coefficient between pulse amplitude (AMP) and intracranial pressure (ICP) (1 mmHg = 0.133 kPa).

### 4.3 Correlation of the spindle wave with outcome

**FIGURE 2:**
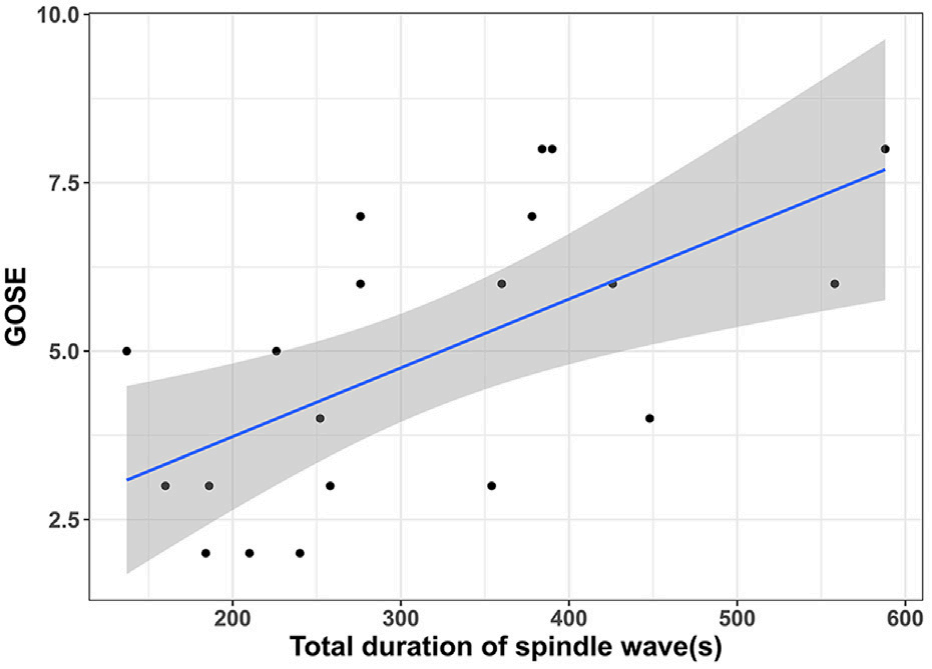
| Relationship between the total duration of spindle wave appearance and the prognosis of TBI patients showed that the longer the total duration of spindle wave appearance, the higher the patient’s Glasgow Outcome Scale-Extended (GOSE) score at 6 months.

In the spindle wave group, we continued to analyze the relationship between spindle wave parameters and the prognosis of TBI patients. We found a significant correlation between the total duration of spindle wave appearance and GOSE at 6 months (r = 0.62, *p* = 0.004, Figure 2). No correlation was found between the number of segments of spindle waves and 6-month GOSE (*p* = 0.41). No relationship was found between the duration of each segment of spindle waves and 6-month GOSE (*p* = 0.66).

### 4.4 Relationship between the appearance of the spindle wave and ICP waveform parameters

**FIGURE 3:**
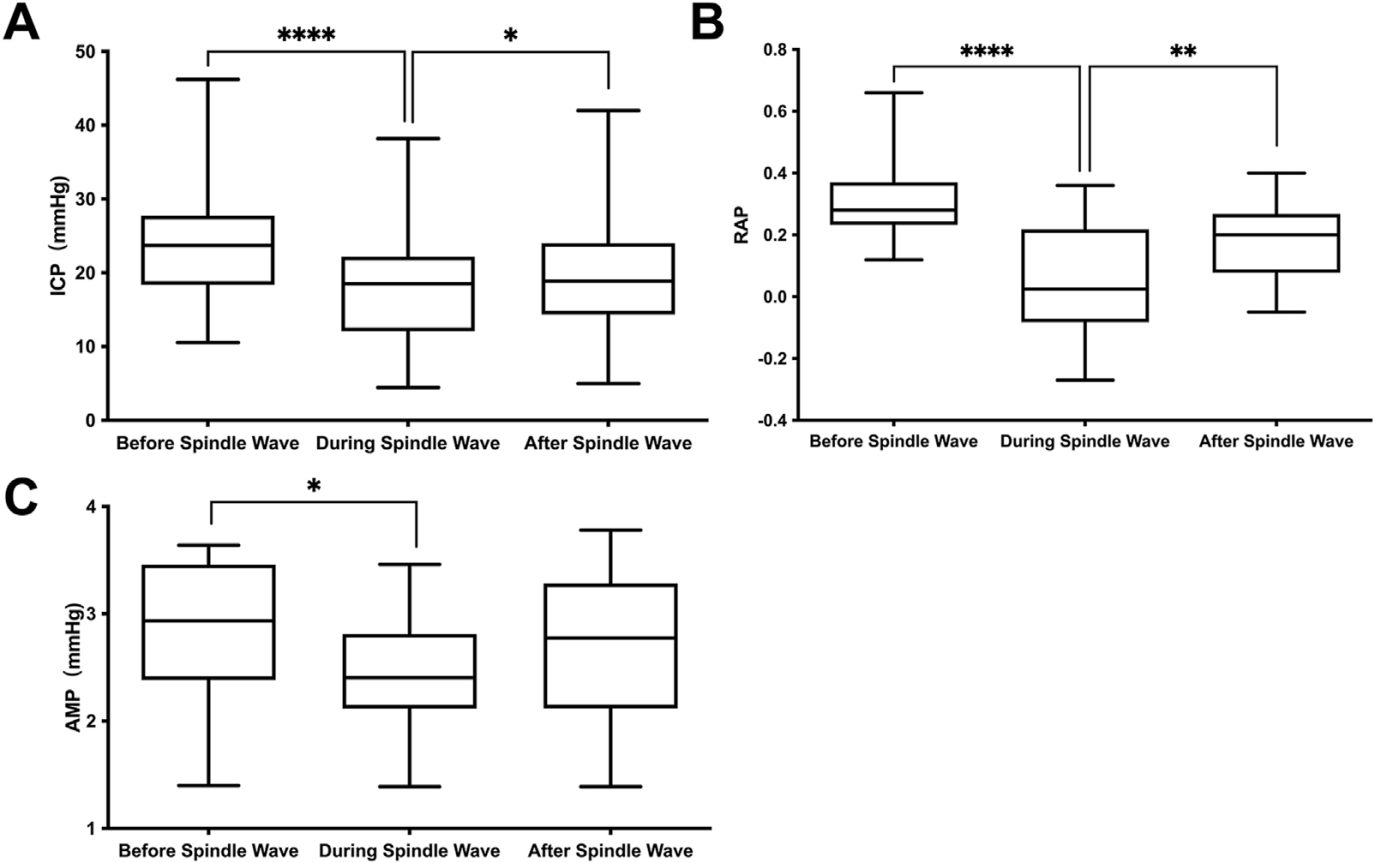
Relationship between the appearance of the spindle wave and ICP waveform parameters. **(A)** Intracranial pressure (ICP), **(B)** correlation coefficient between AMP and ICP (RAP) and **(C)** ICP pulse amplitude (AMP). *Difference with a confidence level of *p*<0.025.

Based on the significant correlation of spindle waves with the outcome, we further compared the differences of various ICP waveform parameters before, during, and after the spindle wave in the spindle wave group, which were shown in Table 2; Figure 3. The mean ICP value during the spindle wave was significantly lower than that before the appearance and after the disappearance of the spindle wave (*p* < 0.0001, *p* = 0.02, respectively, Figure 3A). RAP during spindle wave was also significantly lower than that before and after spindle wave (*p* < 0.0001, *p* < 0.01, respectively, Figure 3B). Besides, AMP during the spindle wave was significantly lower than before the spindle wave (*p* = 0.015, Figure 3C).

**TABLE 2:** Comparative results of parameters before, during, and after the spindle wave in patients with traumatic brain injury (TBI) (mean ± standard deviation).

Time	ICP (mmHg)	RAP	AMP (mmHg)
Before the appearance of the spindle wave	23.31 ± 7.80	0.31 ± 0.13	2.80 ± 0.68
During the appearance of the spindle wave	18.11 ± 7.67^a^ ^,^ ^b^	0.04 ± 0.18^a^ ^,^ ^b^	2.41 ± 0.57^a^
After the disappearance of the spindle wave	19.86 ± 8.46	0.18 ± 0.12	2.77 ± 0.67

^a^ Means *p* < 0.025 compared with before the appearance of the spindle wave.

^b^ Means *p* < 0.025 compared with after the appearance of the spindle wave; ICP, is intracranial pressure; AMP, is ICP pulse amplitude; RAP, is the correlation coefficient between pulse amplitude (AMP) and intracranial pressure (ICP) (1 mmHg = 0.133 kPa).

## 5 Discussions

Based on the observation of the spindle wave during clinical ICP monitoring in the center, we collected the ICP data prospectively to compare and analyze the clinical significance. Our results show that patients in the spindle wave group had a better prognosis and there was a positive correlation between the accumulated duration of spindle waves during hospitalization and the patient’s 6-month GOSE. In addition, analysis of the alteration in AMP and RAP indexes during the spindle wave also yielded interesting results that deserve further emphasis. These findings support the theory that monitoring ICP signal and waveform parameters in addition to ICP values can provide additional clinical significance (Di Ieva et al., 2013).

It is well known that ICP waveforms are classified into three components, including pulse waveform, respiratory waveform, and slow waveform, while Lundberg et al. described three typical ICP waves, including “A” wave, “B” wave, and “C” wave (Cardoso et al., 1983; Di Ieva et al., 2013). This classification is based on the visual description of the morphological and temporal patterns of slow waves, whose presence may or may not be related to the pathological process. In contrast to the above waves describing the trend of intracranial pressure waves over time, the wave described in this study is a single spindle-like wave formed by increasing and decreasing ICP pulses over a short period (generally within tens of seconds) and cycling for several minutes to form a segment of the spindle wave. This wave has some commonalities with the above waves. They are both distinct shifts between the ICP signal and the baseline wave over some time, although the overall trend of the spindle wave does not change significantly over tens of minutes. Further studies have pointed out that the ICP signal and waveform parameters can be utilized to reflect brain tissue states such as brain compensatory space, intracranial compliance, and brain autonomic regulation (Cardoso et al., 1983; Czosnyka and Pickard, 2004). Lemaire et al. (2002) reviewed in detail the characteristics of slow waves and concluded that a major factor influencing the occurrence and appearance of ICP slow waves is the compliance of the closed space and that analysis of ICP waveforms can be used to assess the intracranial compliance. The theory of intracranial compliance dates back to the Monro-Kellie doctrine in the 18th century (Harary et al., 2018). With a constant total volume of the cranial cavity contents, the pressure is stable, and when the volume of one component increases significantly if it cannot be compensated by reducing the volume of other components, intracranial compliance decreases significantly, causing an increase in ICP. Two indexes that indirectly reflect intracranial compliance by analyzing intracranial pressure waveforms were included in this study.

In the study, all patients received ICP intraventricular sensor insertion. One-third of patients had spindle waves during their hospitalization. The spindle-like wave observed had a shuttle shape with wide middle and narrow ends with an average duration of about 31 min, which can be easily identified from the normal ICP baseline wave. The total duration of the spindle wave during hospitalization for patients in the spindle wave group varied between individuals, ranging from 137 to 588 min. The multi-segmental nature of the appearance of the spindle wave and their persistence over time, as well as the homogeneity of treatment protocol between patients, suggest that the appearance of the spindle waves is a stable physiological phenomenon. Two patients in the spindle wave group showed typical spindle waves without decompressive craniectomy, as well as no difference in the composition of patients with decompressive craniectomy between the two groups, indicating that the appearance of spindle waves was not significantly related to decompressive craniectomy.

The spindle wave group had a better performance in terms of GCS score at discharge, ΔGCS, and 6-month GOSE in the absence of differences in baseline characteristics between groups, suggesting the spindle wave group showed better short-term as well as long-term prognosis. Correlation analysis further demonstrated that the total duration of spindle waves was positively associated with the long-term prognosis (r = 0.62). These data suggest that patients tend to have a better prognosis when they have had the spindle wave during ICP monitoring, and this effect is enhanced with increasing duration of the spindle wave appearance. There was no significant difference in mean ICP value between groups, suggesting that the presence of spindle waves may not be associated with a higher or lower ICP. This partly confirms the view of (Chesnut et al., 2012) that ICP values sometimes do not accurately reflect prognosis in clinical practice. Meanwhile, this suggests that the baseline levels of ICP were consistent between groups. The difference in RAP between groups was significant, indicating that the spindle wave group already exhibited a higher cerebrospinal compensatory reserve during hospitalization.

In the spindle wave group, there was a significant decrease in ICP during the spindle wave compared to before its appearance. Compared with the rapid and significant increase in ICP accompanied by the plateau wave (Czosnyka and Pickard, 2004), the change in ICP during the spindle wave was small but significant. Meanwhile, the ICP waveform parameters, AMP and RAP, were also significantly reduced when the spindle wave appeared, indicating higher intracranial compliance when spindle waves appeared, which may explain the decrease in ICP. The current mainstream view on methods to measure intracranial compliance is based on indexes such as AMP (Eide and Sorteberg, 2007; Eide et al., 2011; Ocamoto et al., 2021), RAP (Czosnyka et al., 2001; Kim et al., 2009), and the rising slope of the ICP pulse waveform (Robertson et al., 1989), three indirect measurement methods based on ICP waveform analysis. Howells et al. further evaluated and compared these three metrics as actual clinical measures of intracranial compliance in a range of TBI patients and found that AMP performed better and easier than the other two metrics (Howells et al., 2012). Significantly, the presence of the spindle wave was followed by a consistent performance of AMP and RAP. The decrease in AMP reflects an increase in cerebrospinal fluid reserve capacity. The RAP index decreased and the mean value remained near 0, suggesting improved pressure-volume reserve. This effect has been reflected in the between-group comparisons of the RAP index. Thus, the improved intracranial compliance at the time of the spindle wave may partially explain why the spindle wave group would have a better prognosis.

Based on the finding that spindle waves were associated with a better prognosis in TBI patients, the improved intracranial compliance during the presence of spindle waves that we further found is clinically significant. There have also been many recent studies on the relationship between intracranial compliance and prognosis. Kazimierska et al. (2021) used ICP pulse waveform combined with RAP index to analyze the prognosis of TBI patients, suggesting that brain compliance analysis can be used to assess the intracranial status of patients together with standard mean ICP monitoring. Brasil et al. (2021) found that intracranial compliance combined with cerebrovascular hemodynamic indicators can accurately predict early adverse outcomes in critically ill patients with COVID-19. These studies suggest that analysis of intracranial compliance may help clinical decision-makers make better judgments about a patient’s intracranial status and even patient prognosis.

The presence of a slow oscillatory component in the ICP signal is well established, and the cause is either vascular-derived or respiratory-derived factors (Newell et al., 2022). For example, B waves also exhibit regular repetitive ICP oscillations with a fixed period (Spiegelberg et al., 2016), which are similar to spindle waves in our study. However, their frequencies are distinctly different, and the mechanism that causes the rapid increase and decrease of ICP amplitude over time is unknown. The regular cycles of amplitude increase and decrease suggest that there is a feedback mechanism behind this phenomenon. These mechanisms need to be further investigated.

Overall, the spindle wave we observed was associated with a better prognosis in patients with TBI. We found that the presence of the spindle wave was accompanied by a dramatic and statistically significant improvement of the patient’s ICP value and ICP waveform parameters reflecting intracranial compliance. These findings may partly explain the improved patient outcomes. However, the present study has some limitations. Firstly, the sample size was small and from a single center, and more clinical cases need to be collected as well as a multi-center collaboration for further analysis. Secondly, because the study was an exploratory study, we did not place specific restrictions on population heterogeneity in terms of age and admission GCS score. Finally, the reason for the occurrence of the spindle wave remains unknown, and if the high amplitude-sleep spindle waves observed in EEGs are due to a higher synchronization of neuronal firing (Normand et al., 2016), the completely different mechanism of the appearance of spindle waves in ICP monitoring in this study requires further investigation.

## 6 Conclusion

In summary, this study observed and analyzed the spindle wave by prospectively monitoring the intracranial pressure (ICP) in 60 patients with traumatic brain injury (TBI) and found the spindle wave group had a better prognosis, and the total duration of spindle waves was positively correlated with 6-month GOSE. Meanwhile, the physiological parameters including the ICP, AMP, and RAP showed significant improvement during the spindle wave. These may explain the improved prognosis of patients, but the specific mechanism remains to be further studied.

## Data Availability

The original contributions presented in the study are included in the article/supplementary material, further inquiries can be directed to the corresponding authors.
